# Traumatic brain injury in the elderly after a skiing accident: A retrospective cohort study in a level 1 emergency department in Switzerland

**DOI:** 10.1371/journal.pone.0273168

**Published:** 2022-08-17

**Authors:** Alberto Consuegra, Katharina Lutz, Aristomenis K. Exadaktylos, Werner J. Z’Graggen, Rebecca M. Hasler

**Affiliations:** 1 Department of Neurosurgery, University of Bern, Bern University Hospital, Bern, Switzerland; 2 Department of Emergency Medicine, University of Bern, Bern University Hospital, Bern, Switzerland; University Hospital Zurich, SWITZERLAND

## Abstract

**Background:**

Skiing is a very popular sport worldwide, with increasing trends over the past decades. This study aimed to evaluate the importance of traumatic brain injury (TBI), especially in the elderly, after a ski accident, and to describe its short-term repercussions.

**Methodology:**

Patients were analyzed who were admitted to our neurotrauma center from 2012–2018 after a head trauma while skiing. Three different age groups were differentiated and analyzed for the severity of TBI depending on the initial Glasgow Coma Scale as the primary outcome and as secondary outcomes need and type of surgery, Glasgow Outcome Score, preexisting use of anticoagulant or antiplatelet drugs, time to presentation, and pattern of brain injury. TBI severity was adjusted to the time to initial medical consultation.

**Results:**

No significant difference in TBI severity was found when comparing the middle (>29–54) and older (≥54) age groups to the reference group <30 years (OR:0.45, p = 0.127; OR:0.46, p = 0.17). Acute subdural hemorrhage was present in 21.2% of the ≥55 group and 14.5% of the 30–54 age group, compared to 12.8% of the youngest group (p = <0.001). Overall, 39.4% of the patients in the ≥55 group and 8.1% of the 30–54 age group presented with chronic subdural hemorrhage, whereas none of the youngest patients did (p = <0.001).

**Conclusion:**

No differences were observed in terms of TBI severity between age groups after acute trauma. Nonetheless, a different pattern of head injury after TBI in older patients was demonstrated. Accordingly, the management differs for these TBIs compared to those of younger patients.

## Introduction

### Skiing accidents

Skiing is a very popular sport worldwide for most of the active population, with increasing trends over the past decades [1–3]. Swiss national statistical data show that the number of older people with a very active lifestyle is following this dynamic as well, with skiing being one of the four favorite sports in the country. Skiing, together with soccer, is also the sport generating the most accidents [4, 5]. In economic terms, Swiss national data have shown that ski and snowboard accidents generate mean costs of 240.4 Mio CHF (mean for 2013–2017) per year [6].

### Prevention

Even though the use of helmets has become increasingly popular among skiers of all ages, traumatic brain injury (TBI) remains associated with up to 10% of all skiing accidents. In addition, more than 85% of affected people, including all types of skiing injuries, needed medical consultation directly after their trauma. In the skiing season of 2017/2018, 30% of the referred skiing and snowboarding accidents involved individuals older than 50 years [7].

Although skiing trauma is a well-acknowledged entity, very limited information is available about the sequela of TBI after skiing accidents in the older population. Given an increasingly active elderly population, information about the long-term repercussions of a skiing accident TBI is important.

### Age differences

The elderly form a special population group due to their anatomical, social, and medical particularities.

Aging is widely known to predispose a person to anatomical changes in the brain, including cerebral atrophy. This process is rapidly accelerated in the population older than 60 years [8]. At that age, adherence of the dura mater also increases, facilitating the tearing of bridge veins and promoting the formation of subdural hemorrhages. Furthermore, the prescription of antiplatelet drugs and oral anticoagulants (OACs) increases, especially in the elderly, due to a well-demonstrated benefit in the primary and secondary prevention of vascular disease [9]. In parallel, a higher prevalence of intracerebral hemorrhage (ICH) and subdural hematoma (SDH) has been demonstrated in this patient group [10, 11].

An increased incidence of neurosurgical diseases such as chronic subdural hematoma (cSDH) also exists in the elderly. Two studies, one from Finland and one from Japan, including 63,358 patients demonstrate this fact [12, 13]. The different brain pathology incidences in older patients, such as cSDH, could also lead to differences according to the timing of hospital admission, e.g., older patients with cSDH tend to present later to hospital.

In addition, some of the recent international data suggest a poorer outcome after head trauma in the elderly population compared to younger age groups [14, 15]. Nevertheless, some data also show a positive outcome trend in the elderly with head trauma after receiving modern intensive care treatment [16].

Our hospital is the neurotrauma center of reference for one of the biggest ski regions in the country, including an area of around 1.7 million potential patients. Therefore, we decided to retrospectively analyze the characteristics after head trauma of patients admitted to a “Swiss level I trauma center” after a skiing TBI, focusing mainly on the elderly population.

This study aimed to evaluate the importance of TBI, especially in the elderly, after a ski accident and to describe its short-term repercussions.

## Methods

A retrospective cohort study was conducted of patients admitted to our Swiss level I emergency department (ED) after a skiing accident. We differentiated three age groups and analyzed the severity of TBI as the primary outcome and as secondary outcomes the need and type of surgery, the Rotterdam CT score, the Glasgow Outcome Score (GOS), the preexisting use of OACs or antiplatelet drugs, the time to presentation at our ED, and the pattern of brain injury.

The inclusion criteria were patients ≥16 years who were admitted to the ED of the University Hospital Bern, Switzerland, between 12/2012 and 12/2018, after TBI as a result of a skiing trauma. We included patients admitted either directly after the accident or up to 3 months after trauma. The exclusion criteria were patients presenting with an isolated facial trauma (meaning facial bone fracture or superficial face wounds without signs of TBI), or any other skiing trauma to other body regions if no sign of TBI was evident. In patients who were not directly admitted but with a latency (of up to 3 months), hospital admission reports were carefully checked for any other accidents in between. However, we did not have to exclude any patients for this reason. Additionally, patients with head trauma not resulting directly from skiing (e.g., trauma from skiing lifts or parking areas in the ski regions) were excluded.

### Data preparation

Patients were grouped by age into three groups (16–29, 30–54 and ≥55 years). Traditionally, the age threshold for elderly patients starts from age 65. In this study, we chose a lower threshold from 55 years of age to achieve a more representative distribution. Similar to our study, this lower threshold has been used by different authors describing TBI patients in the past [17, 18].

TBI severity was analyzed according to the documented Glasgow Coma Scale (GCS) at the site of the accident or if not available on admission for those presenting with acute trauma: 3–8 (severe), 9–12 (moderate), or 13–15 (mild) points. The GOS was determined at the time of discharge from our hospital [19]. The pattern of brain injury was analyzed according to the emergency CT scan (intracerebral, subdural, epidural, or subarachnoid hemorrhages, any intracranial hemorrhage with or without fractures). Furthermore, the type of performed surgery (burr hole, craniotomy, hemicraniectomy, external ventricular drainage catheter [EVD], or other intracranial pressure [ICP] monitoring probes), time to admission to the ED after trauma (before 24 h or after 24 h), whether Rotterdam CT scores were determined for acute trauma (<24 h), and the use of OACs or antiplatelet agents (yes/no) were analyzed.

### Statistics

#### Primary and secondary outcomes

The primary outcome was the TBI severity according to the GCS. In addition, we analyzed as secondary outcomes the need and type of surgery, the GOS, the use of antiplatelet or anticoagulant drugs, the time to presentation at our ED, and the pattern of brain injury.

The descriptive statistics included the number and percentage for categorical parameters and either mean (SD) or median and interquartile range (IQR) for continuous parameters, such as score values. Significance tests, such as the rank sum, Fisher’s exact, or chi-squared tests, provided the corresponding p-values.

Logistic regression models were performed to study the effect of the various parameters on the three age groups. Patients aged <30 were considered the reference group. For GOS, a multiple logistic regression was performed with "5, good recovery" as the reference level. Rotterdam scores were considered Poisson distributed, allowing for overdispersion in the regression models. All regression models were additionally adjusted for sex. Odds ratios with the corresponding 95% confidence intervals (CI) and p-values were calculated. A p-value <0.05 was considered statistically significant. An adjustment of the significance level for multiple comparisons was omitted because of the descriptive nature of the study.

All analyses were performed with the statistical program R version 3.5.1. [20].

### Ethics

All participants provided written general consent. The project was approved by the local ethics committee “Kantonale Ethikkommission für die Forschung in Bern” on 18/08/2020 with the project ID number 2020–01116.

## Results

**Fig 1:**
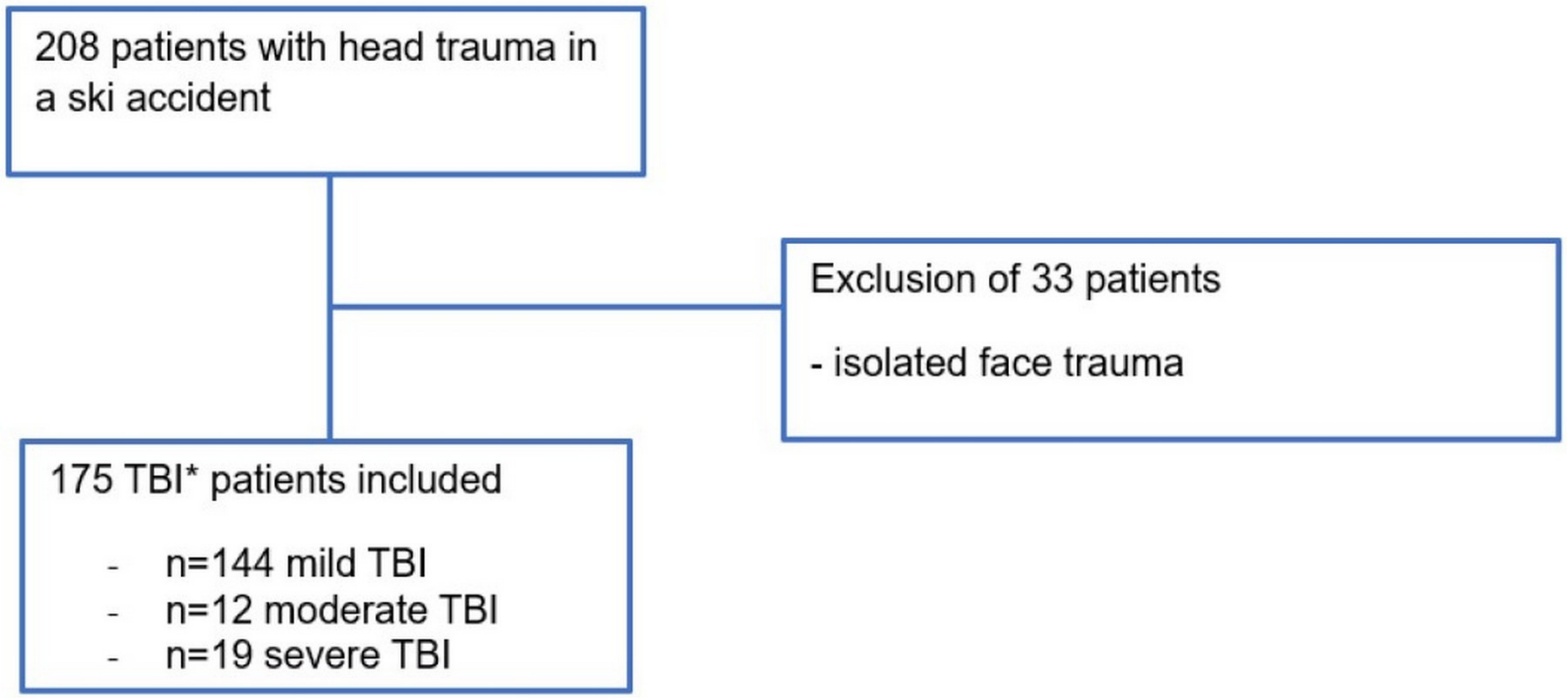
Flow chart of patient inclusion and exclusion. *TBI = Traumatic Brain Injury.

As displayed in Fig 1, a total of 208 patients were admitted to our ED after head trauma related to a ski accident in our 7-year study (2012 to 2018). After applying our exclusion criteria, 175 patients with TBI after a skiing accident were analyzed.

### Patient characteristics

Table 1 displays the characteristics of the included patients. The median age was 49 years (IQR 62.5–28); 68.0% were men (n = 119) and 32.0% were women (n = 56). Overall, 26.9% of all patients were included in the age group <30 years (n = 47), 35.4% in the middle group from 30 to 54 years old (n = 62), and 37.7% in the group ≥55 years old (n = 66). The median GCS was 15 (IQR 15–14 = 1).

**Table 1:** Patient characteristics.

	Age			p-value	Total
	<30	30–54	>54		
**No. patients (%)**	47 (26.9)	62 (35.4)	66 (37.7)	0.076	175
**Sex**					
Male n (%)	31 (66)	40 (64.5)	48 (72.7)	0.57	119 (68.0)
Female n (%)	16 (34)	22 (35.5)	18 (27.3)		56 (32.0)
**GCS median (IQR)**	14 (9.5,15)	15 (14,15)	15 (15,15)	<0.001	15 (14,15)
**GCS at admission**					
13–15 n (%)	33 (70.2)	53 (85.5)	58 (87.9)	0.060	144 (82.3)
9–12 n (%)	4 (8.5)	3 (4.8)	5 (7.6)		12 (6.9)
3–8 n (%)	10 (21.3)	6 (9.7)	3 (4.5)		19 (10.9)
**Neurosurgical intervention**					
Yes n (%)	4 (8.5)	10 (16.1)	32 (48.5)	<0.001	46 (26.3)
No n (%)	43 (91.5)	52 (83.9)	34 (51.5)		129 (73.7)
**Time to admission at the ED**					
<24 h n (%)	39 (83)	40 (64.5)	29 (43.9)	<0.001	108 (61.7)
>24 h n (%)	8 (17)	22 (35.5)	37 (56.1)		67 (38.3)
**GOS**					
1 (death) n (%)	2 (4.3)	4 (6.5)	0	0.24	6 (3.4)
2 (persistent vegetative state) n (%)	0	0	0		0
3 (severe disability) n (%)	1 (2.1)	0	2 (3)		3 (1.6)
4 (moderate disability) n (%)	1 (2.1)	2 (3.2)	1 (1.5)		4 (2.3)
5 (no or low disability) n (%)	40 (85.1)	54 (87.1)	62 (93.9)		156 (89.1)
Missing data (%)	3 (6.4)	2 (3.2)	1 (1.5)		6 (3.4)
**Anticoagulation**					
Yes n (%)	0	7 (11.3)	18 (27.3)	<0.001	25 (14.3)
No n (%)	47 (100)	52 (83.9)	47 (72.2)		146 (83.4)
Missing data (%)	0	3 (4.8)	1 (1.5)		4 (2.3)
**Pattern of injury** *					
No bleed n (%)	28 (59.6)	42 (67.7)	19 (28.8)	<0.001	89 (50.9)
Subarachnoid hemorrhage n (%)	9 (19.1)	4 (6.5)	11 (16.7)		24 (13.7)
Intracerebral hemorrhage n (%)	6 (12.8)	6 (9.7)	4 (6.1)		16 (9.1)
Chronic subdural hemorrhage n (%)	0	5 (8.1)	26 (39.4)		31 (17,1)**
Acute subdural hemorrhage n (%)	6 (12.8)	9 (14.5)	14 (21.2)		29 (16.0)
Epidural hemorrhage n (%)	0	0	1 (1.5)		1 (0.6)
Shearing injuries n (%)	9 (19.1)	1 (1.6)	3 (4.5)		13 (7.4)
Calvarial fracture n (%)	2 (4.3)	1 (1.6)	1 (1.5)		4 (2.3)
Skull base fracture n (%)	3 (6.4)	2 (3.2)	0		5 (2.9)
**Type of surgery**					
Burr hole n (%)	0	3 (4.8)	23 (34.8)	<0.001	26 (14.9)
Craniotomy n (%)	0	3 (4.8)	7 (10.6)		10 (5.7)
Intracranial monitoring n (%)	4 (8.5)	1 (1.6)	1 (1.5)		6 (3.4)
Hemicraniectomy n (%)	0	3 (4.8)	1 (1.5)		4 (2.3)
No operation n (%)	43 (91.5)	52 (83.9)	34 (51.5)		129 (73.7)

TBI: Traumatic Brain Injury, GCS: Glasgow Coma Scale, GOS: Glasgow Outcome Scale, ED: Emergency Department, IQR: Interquartile Range

*14.9% (n = 26) of the analyzed individuals presented a combined injury pattern.

**8 out of 31 patients with cSDH (25.8%) were on anticoagulation.

Regarding the type of brain injury, the majority (50.9%, n = 89) presented with signs or symptoms of TBI without any additional radiological findings. This was only the case in 28.8% (n = 19) of the ≥55 years group.

Only one patient (0.6%) experienced an epidural hemorrhage, whereas 13.7% (n = 24) had a traumatic subarachnoid hemorrhage and 9.1% (n = 16) an ICH. Moreover, 14.9% (n = 26) of the analyzed individuals presented a combined injury pattern.

**Fig 2:**
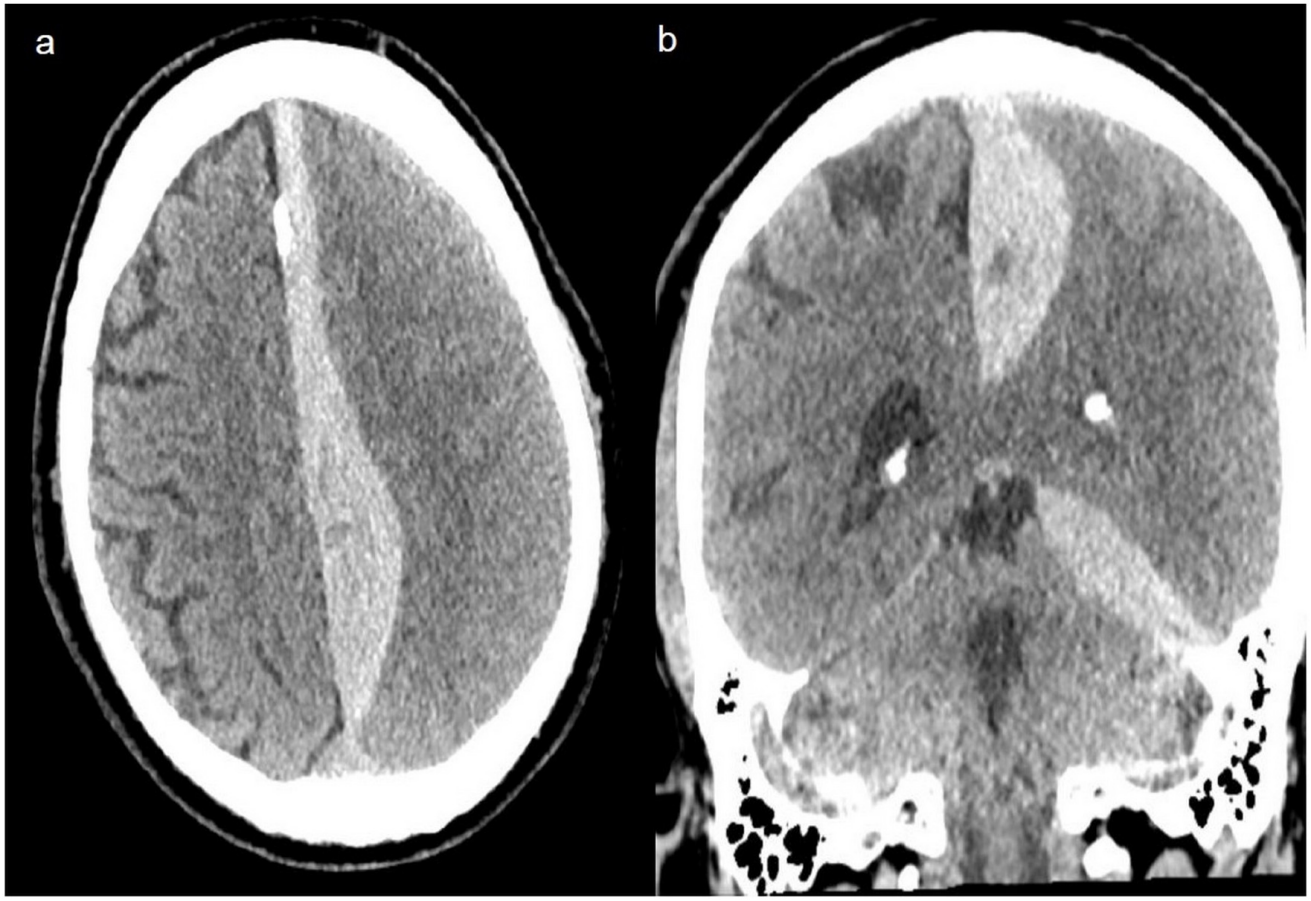
Case illustration. Patient 1. A 64-year-old male patient after a skiing accident with head trauma under anticoagulation (phenprocoumon and acetylsalicylic acid). He presented with an acute subdural hematoma with an interhemispheric compressive effect. The GCS at hospital arrival was 15 points, with right leg paralysis (M4/5). CT scan with a) axial and b) coronal view.

**Fig 3:**
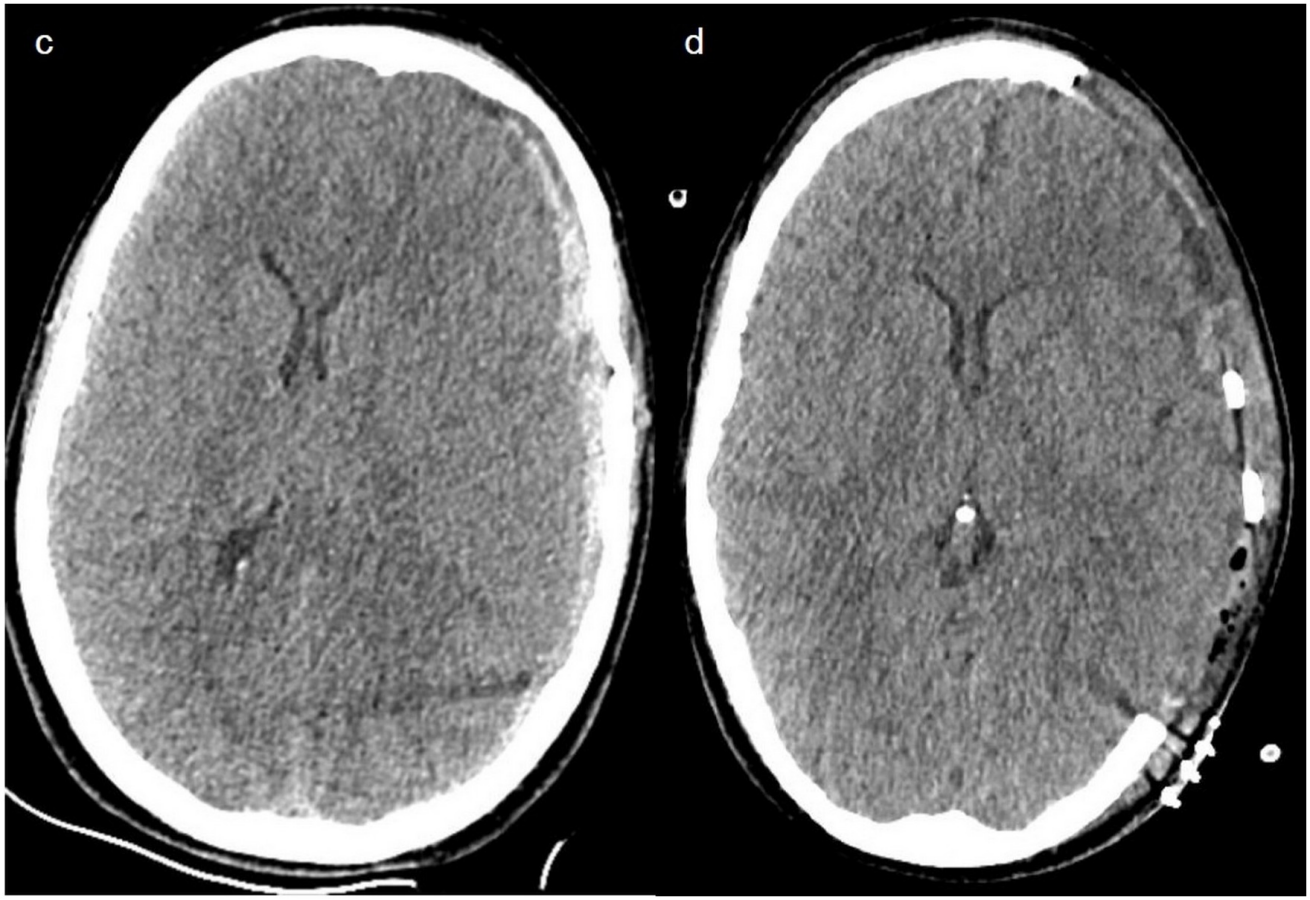
Case illustration. Patient 2. A 39-year-old female patient after a skiing accident with head trauma. The GCS at rescue team arrival was 12 points, with a rapid decrease to GCS of 5 points and development of a left anisocoria c) In the CT scan, we observe an acute subdural hematoma with an important midline shift. She underwent a decompressive hemicraniectomy and left hospital 2 weeks later with a GCS of 15. d) Postoperative CT scan.

The frequency of an acute subdural hematoma (aSDH), as displayed with a CT scan of two example patients in Figs 2 and 3, was higher in the ≥55 years group with 21.2% (n = 14) than in the younger patients. In the youngest age group, the frequency of an aSDH was 12.8% (n = 6), and in the middle age group, it was 14.5% (n = 9).

Overall, 39.4% of the patients (n = 26) in the oldest group presented with cSDH, whereas no patients in the youngest group had this diagnosis.

The surgical management of the different age groups was also analyzed. Overall, 73.7% (n = 129) of patients did not undergo surgery. However, these results differed by age group, with 91.5% (n = 43) of the youngest group not needing surgical intervention, whereas 48.5% (n = 32) of the oldest age group underwent surgery. No other types of interventions were needed. By contrast, only 51.5% (n = 34) in the oldest group received conservative management; 34.8% (n = 23) and 10.6% (n = 7) underwent burr hole and craniotomy with hematoma evacuation, respectively. In the middle age group, 83.9% (n = 52) were likewise managed conservatively, with 4.8% (n = 3) undergoing a decompressive hemicraniectomy.

Overall, 89.1% (n = 156) had a maximal GOS of 5 with no or minor disability at discharge, while 3.4% (n = 6) had a GOS of 1 (death).

The time to medical consultation after trauma revealed a significant delay for the oldest group of patients: 56.1% (n = 37) of these patients presented later than 24 hours after the TBI, whereas only 17% (n = 8) of the youngest patients were admitted to the ED with a delay longer than 24 h after trauma. In total, 61.7% (n = 108) of all patients arrived at the ED within the first 24 h after trauma.

### Logistic regression analysis

The main analysis compared TBI severity between the three age groups. Only the acute cases were included in this analysis. For this, TBI severity was adjusted to the time to initial medical consultation (admission to ED <24 h after trauma). No significant difference was found when comparing the middle age group to the reference group <30 years (OR: 0.45, 95% CI: 0.16–1.25, p = 0.127) or the older group (OR: 0.46, 95% CI: 0.15–1.4, p = 0.17).

In the age-adjusted model with neurosurgical intervention as the dependent variable, the oldest group had significantly higher odds for surgery compared to the youngest group (OR: 9.4, 95% CI: 3.0–29.5, p<0.001). If adjusting the analysis to the time of admission before 24 h after trauma, the significance disappeared (OR: 0.3, 95% CI: 0.03–2.85, p = 0.294).

For the GOS, we took a reference value of 5 and compared the middle age group to the youngest but found no significant difference (OR: 1.48, 95% CI: 0.13–17.28, p = 0.754). The same was true if the oldest group was compared to the youngest (OR:0.54, 95% CI: 0.0.3–8.95, p = 0.664). We also found no significance in terms of outcome if adjusting the data to the time of admission.

The Rotterdam CT score for patients presenting within the first 24 h after trauma was also analyzed. No statistical significance was observed when analyzing the distribution between the three age groups using a linear model adjusted for sex and using the youngest group as a reference. The group from 30–54 years had a geometric mean ratio (GMR) of 0.968 (CI 95%: 0.836–1.121, p = 0.664). The group over 54 years old had a GMR of 0.938 (95% CI: 0.799–1.099, p = 0.431).

Patients in the youngest age group significantly more often presented early (<24 h) at the ED compared to older age groups (age 30–54 years: OR: 0.36, 95% CI: 0.14–0.92, p = 0.033; age ≥55 years: OR: 0.16, 95% CI: 0.07–0.4, p = <0.001).

## Discussion

In our analysis, no significant differences were shown between the different age groups in terms of experiencing a severe TBI in the acute scenario. Furthermore, no significant difference was found between age groups when considering the need for surgical intervention in the acute scenario and likewise for the short-term outcome measured by GOS at discharge. Conversely, higher ORs were observed for the older patients to present to the ED with a delay of days to months after the trauma.

### Severity of TBI and intracranial injury patterns

The results of this study showed no differences in the frequency of severe TBI between the age groups. Despite these data, younger patients were more likely to present no intracranial hemorrhage after TBI. These data are similar to those in the literature and could be explained by many different factors, such as the use of anticoagulant and antiplatelet drugs or the morphological changes in more advanced life stages [14, 15]. Furthermore, cSDH was only found in the middle and older groups. Younger patients may have more other injury patterns such as diffuse axonal injuries (shearing injuries), which could have been underdetected in our analysis due to their low specificity in CT scans [21]. Other studies evaluating older patients (≥65 years) after acute trauma showed a frequency for aSDH of 28.9–45.5% compared to 21.2% in our study [10, 15]. The lower frequency of aSDH in our study could be related to different factors such as the lower age threshold for our elderly group (55 years) and the common use of helmets while skiing nowadays.

As well as the aging process related to brain atrophy facilitating the formation of a chronic subdural hematoma [12, 13, 22, 23], it could contribute to the expansion of other intracranial hemorrhages. Thus, even larger volumes of intracranial hemorrhages could be tolerated longer in the elderly before neurological decline occurs.

### Surgical intervention

We observed higher ORs to undergo neurosurgical intervention for the older patients when analyzing all data independent of the time of admission. However, no significant difference was observed when adjusting the analysis for early admission <24 h after trauma. This finding is similar to other studies [10, 24]. A possible explanation for our older group presenting such a high percentage of surgical interventions could be that it reflects the high number of older patients attending the ED with a significant delay and presenting with cSDH as a late complication of head trauma. Those injuries required hematoma evacuation either with burr hole trepanation or a craniotomy.

Previous data have also described poorer outcomes for elderly patients undergoing surgical intervention after head trauma, with a mortality of 32.9%. In that study, patients received neither craniotomy for hematoma evacuation nor craniectomy for the treatment of refractory brain edema [25]. The cohort was significantly older than ours, starting at 65 years of age, and only consisted of patients with acute trauma. Furthermore, the surgical techniques used were more invasive than in most of our cases.

### Time to admission to ED

Higher odds were demonstrated for a delayed presentation at the ED for the older patients. A reasonable explanation could lie in the pattern of injuries demonstrated in the different age groups, confirming our initial assumption. We observed a higher percentage of older patients presenting with cSDH. This condition could be considered a medium-term complication following head trauma, rather than a direct result of trauma.

The presumption that older patients could present an increased tolerance to intracranial masses and the different types of injury patterns due to intracranial anatomical changes would also support these data.

### Anticoagulation

In this study, 14.6% of the included patients were taking OACs or antiplatelet agents, but none were in the youngest group. The highest number of patients using this kind of drug belonged to the oldest study group, which was also the group with more patients presenting with intracranial hemorrhage. This finding could indirectly support the results from various other authors who have already demonstrated an association between the use of OACs and antiplatelet agents and a higher incidence of intracranial hemorrhage and poorer outcomes after TBI [10, 11].

### Glasgow outcome score

Most of our patients (89.1%) were classified with GOS 5 (no or low disability). No statistical significance was observed between age groups in terms of prognosis. This finding was also robust when only analyzing acute trauma patients. The overall mortality was 3.4%, meaning a total of six patients. No patients in the oldest group died.

Comparing our results to those in the literature, other research work about TBI in elderly patients has presented less favorable neurological outcomes. Their percentages of patients with a GOS of 5 ranged from 51% to 78% [10, 16]. Furthermore, in contrast to us, other studies have shown higher ORs for older patients to present with an unfavorable outcome compared to younger patients [26]. An explanation for this finding might be that our study included many patients presenting late (>24 h) to the ED, whereas other studies only included acute trauma.

The good GOS at discharge for the older patients presenting with cSDH is also supported by the literature for older patients with cSDH undergoing surgery [27, 28].

### Strengths and limitations

A strength of our study is that our hospital is the only reference head trauma center in one of the most important ski regions of Switzerland. Moreover, a detailed neurosurgical description for TBI in a skiing scenario is missing, so this study represents one of the first papers in this field. Conversely, this could have been an important bias in our patient selection. First, patients who were involved in less severe accidents may have been referred to peripheral hospitals for workup and treatment. Another limitation is our GOS determination at discharge, meaning that no follow-ups were considered.

### Implications

The increasing number of older people being active is a positive way of preserving a healthy society. However, as we showed in our study, increasing age is associated with different patterns of injuries compared with the younger population. The late presentation of symptoms in the older population after head trauma and the higher probability of developing post-traumatic complications, such as cSDH, underlines the importance of social education in terms of symptom presentation and the importance of early medical examinations. A cSDH is a perfectly treatable disease, even in the oldest population. Furthermore, different authors have already shown poorer outcomes for patients presenting with cSDH and lower GCS at admission [29–31]. Therefore, an early medical presentation could facilitate the management and improve the outcome.

## Conclusion

Even though we observed no difference in TBI severity between age groups in the acute scenario, the injury pattern in older skiers seemed to differ from that of the younger population. Likewise, the management of the different age groups seemed to differ when considering the middle-term considerations. A late medical presentation and diagnosis of older patients could adversely affect proper ambulatory management. Although cSDH might not be detected in all cases on the initial CT scan, knowing of these patients, they could have been invited for a follow-up visit or CT scan. Education and instruction of older skiers and primary care physicians could help facilitate early diagnosis and proper management.

## Supporting information

S1 Table Time to admission at the emergency department (ED). (DOCX)

S2 Table Logistic regression analysis with neurosurgical intervention and age group. (DOCX)
