# Supplementary material for: Traumatic brain injury in the elderly after a skiing accident: A retrospective cohort study in a level 1 emergency department in Switzerland
Source: PLoS One. 2022 Aug 17;17(8):e0273168. doi: 10.1371/journal.pone.0273168 (PMC9384986; doi:10.1371/journal.pone.0273168)
Supplement: S1 Table — (DOCX) [file pone.0273168.s001.docx]

S1 Supplementary Table. Time to admission at the emergency department (ED).

| **Time to admission at the ED** | **Age** | | | **Total** |
| --- | --- | --- | --- | --- |
|  | **>30** | **30–54** | **>54** |  |
| <24 h (**n [%]**) | 39 (36.1) | 43 (39.8) | 26 (24.1) | 108 (61.7) |
| 1–7 days (**n [%]**) | 8 (25.8) | 14 (45.2) | 9 (29.0) | 31 (17.7) |
| 8–29 days (**n [%]**) | 1 (7.7) | 4 (30.8) | 8 (61.5) | 13 (7.4) |
| >30 days (**n [%]**) | 0 | 3 (13.0) | 20 (87.0) | 23 (13.1) |
