# Supplementary material for: Traumatic brain injury in the elderly after a skiing accident: A retrospective cohort study in a level 1 emergency department in Switzerland
Source: PLoS One. 2022 Aug 17;17(8):e0273168. doi: 10.1371/journal.pone.0273168 (PMC9384986; doi:10.1371/journal.pone.0273168)
Supplement: S2 Table — (DOCX) [file pone.0273168.s002.docx]

S2 Supplementary Table. Logistic regression analysis with neurosurgical intervention (dependent variable) and age group.

|  | Crude OR | 95% CI | Adjusted* OR | 95% CI | P-value |
| --- | --- | --- | --- | --- | --- |
| Reference: Age <30 years | | | | | |
| Age 30–54 | 1.83 | (0.53, 6.34) | 1.85 | (0.53, 6.47) | 0.333 |
| Age >54 | 9.52 | (3.07, 29.56) | 9.44 | (3.02, 29.49) | <0.001 |
| Admitted <24 h after trauma | | | | | |
| Age 30–54 | 0.97 | (0.23, 4.19) | 1.02 | (0.23, 4.46) | 0.976 |
| Age >54 | 0.31 | (0.03, 2.96) | 0.3 | (0.03, 2.85) | 0.294 |

*Adjusted for sex, CI: confidence interval
